# Integrating liquid biopsy and mutational signatures to advance precision oncology

**DOI:** 10.1038/s41698-026-01337-w

**Published:** 2026-02-23

**Authors:** Raquel Carrasco, Kristian Dreij

**Affiliations:** Institute of Environmental Medicine, Karolinska Institutet, Solna, Sweden

**Keywords:** Cancer, Computational biology and bioinformatics, Oncology

## Abstract

The effective application of precision oncology in solid tumors remains challenging due to genetic heterogeneity and the absence of actionable alterations in some cancers. In this review, we discuss the integration of liquid biopsy and mutational signatures as a potential framework to address these limitations by enabling longitudinal detection of mutational processes that arise during tumor development and evolution. Together, these complementary approaches hold substantial promise for enhancing cancer screening, refining diagnosis, and guiding personalized therapeutic strategies, thereby advancing the field of precision oncology.

## Introduction

Cancer is a genetically heterogeneous disease, exhibiting molecular diversity both among patients (intertumor heterogeneity) and within tumor cells of an individual tumor or patient (intratumor heterogeneity)^1^. Intratumor heterogeneity may arise spatially, across different tumor regions, or temporally, as genetic alterations accumulate over time. Consequently, tumors often contain multiple subclonal populations that evolve dynamically, contributing to treatment failure through the development of drug-resistance mechanisms^2,3^.

Precision oncology has emerged as a personalized therapeutic approach aimed at improving treatment efficacy, minimizing adverse effects, and overcoming acquired drug resistance. This strategy relies on comprehensive molecular characterization of each patient’s tumor and integrates diagnostic and therapeutic applications by identifying biomarkers associated with specific cancer subtypes. As a result, precision oncology facilitates the rational development of highly targeted therapies to modulate tumor progression^4–6^. Advances in sequencing and molecular technologies have expanded the clinical use of targeted therapies, which increasingly complement or replace conventional cytotoxic chemotherapy. Numerous agents targeting oncogenic drivers are now approved by the U.S. Food and Drug Administration (FDA) and the European Medicines Agency (EMA) and are used across multiple cancer types. For example, non-small cell lung cancer (NSCLC) patients harboring specific *EGFR* mutations are treated with gefitinib, erlotinib, afatinib, or osimertinib^7^, while patients with bladder cancer carrying *FGFR3* alterations benefit from erdafitinib ^8^.

However, dynamic tumor heterogeneity cannot be adequately captured by standard tissue biopsy, which samples only a small portion of the tumor at a single time point^9^. This constraint represents a major barrier to the effective implementation of precision oncology in solid tumors. To address this limitation, liquid biopsy has emerged as a minimally invasive, repeatable alternative for real-time monitoring of tumor evolution and therapeutic response by detecting tumor-derived mutations at the variant level in cell-free DNA (cfDNA)^10^. Despite its growing relevance, current clinical applications of liquid biopsy remain largely variant-centric and restricted to the detection of predefined actionable mutations. Importantly, actionable alterations are not present in all cancer types or patients, and only a modest number of genes have been validated as cancer drivers or therapeutic targets^11,12^.

In parallel, advances in DNA sequencing technologies have enabled comprehensive, genome-wide characterization of somatic variants accumulated during carcinogenesis, revealing distinctive patterns of somatic mutations known as mutational signatures^13^. These signatures capture the biological processes driving tumor heterogeneity and therapy resistance, offering a process-centric view of tumor evolution that complements variant-level analyses.

**Fig. 1:**
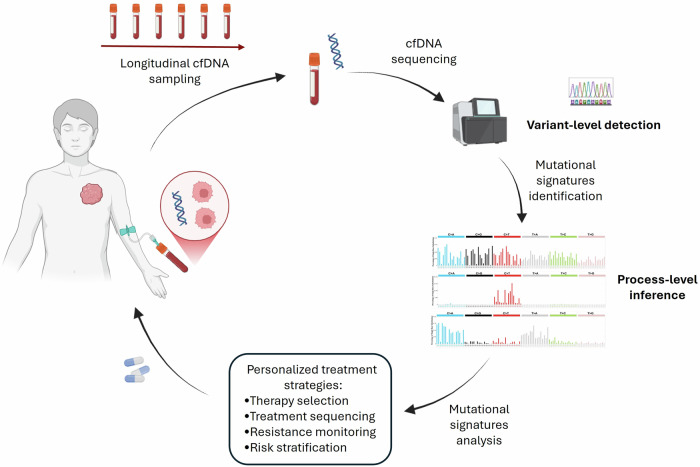
Integrative framework combining liquid biopsy and mutational signatures for process-level tumor monitoring. Longitudinal cell-free DNA (cfDNA) sampling enables repeated, minimally invasive access to tumor-derived DNA released into the circulation. Genome-wide cfDNA sequencing supports both variant-level mutation detection and process-level inference through mutational signature analysis. Mutational signatures, inferred using reference catalogs, such as COSMIC, reflect the biological and environmental processes shaping tumor evolution. Representative mutational signature plots are shown for illustrative purposes and do not represent patient-specific data. Integration of variant-centric and process-centric information may support clinical decision-making, including therapy selection, treatment sequencing, resistance surveillance, and risk stratification. This schematic illustrates a conceptual and translational framework; mutational signature–based assays are not yet clinically approved.

Thus, mutational signatures represent a complementary class of biomarkers that extend the interpretive power of cfDNA analysis beyond individual variants alone. By integrating variant-centric cfDNA profiling with process-level mutational signature analysis, it becomes possible to monitor not only which mutations are present, but also why they arise and how tumors evolve under environmental, therapeutic, and biological pressures (Fig. 1).

In this review, we discuss the complementary roles of two critical tools in modern cancer research—liquid biopsy and mutational signatures. Beyond describing each approach independently, this review's central conceptual advance is to propose their integration as a unified framework for understanding tumor development and evolution at both the variant and process levels. While liquid biopsy enables longitudinal access to tumor-derived material, mutational signatures provide insight into the mutational processes shaping the cancer genome. Together, these approaches offer a powerful strategy to enhance tracking of tumor progression, refine personalized treatment strategies, and ultimately improve clinical management (Fig. 1). This review focuses primarily on solid tumors, where liquid biopsy offers unique advantages over conventional tissue biopsy and where significant unmet clinical challenges remain.

### Liquid biopsy: Concept and clinical implications

The concept of liquid biopsy was first introduced in 2010 with the analysis of circulating tumor cells (CTCs) in the blood of cancer patients^14^, and has since expanded to include circulating tumor DNA (ctDNA), as well as additional tumor-derived components, such as cell-free RNA, extracellular vesicles, and tumor-educated platelets^10^. Although blood is the most commonly used source, circulating biomarkers can also be detected in other body fluids, including urine, cerebrospinal fluid, saliva, and sputum, thereby expanding the applicability of liquid biopsy across diverse clinical contexts.

**Fig. 2:**
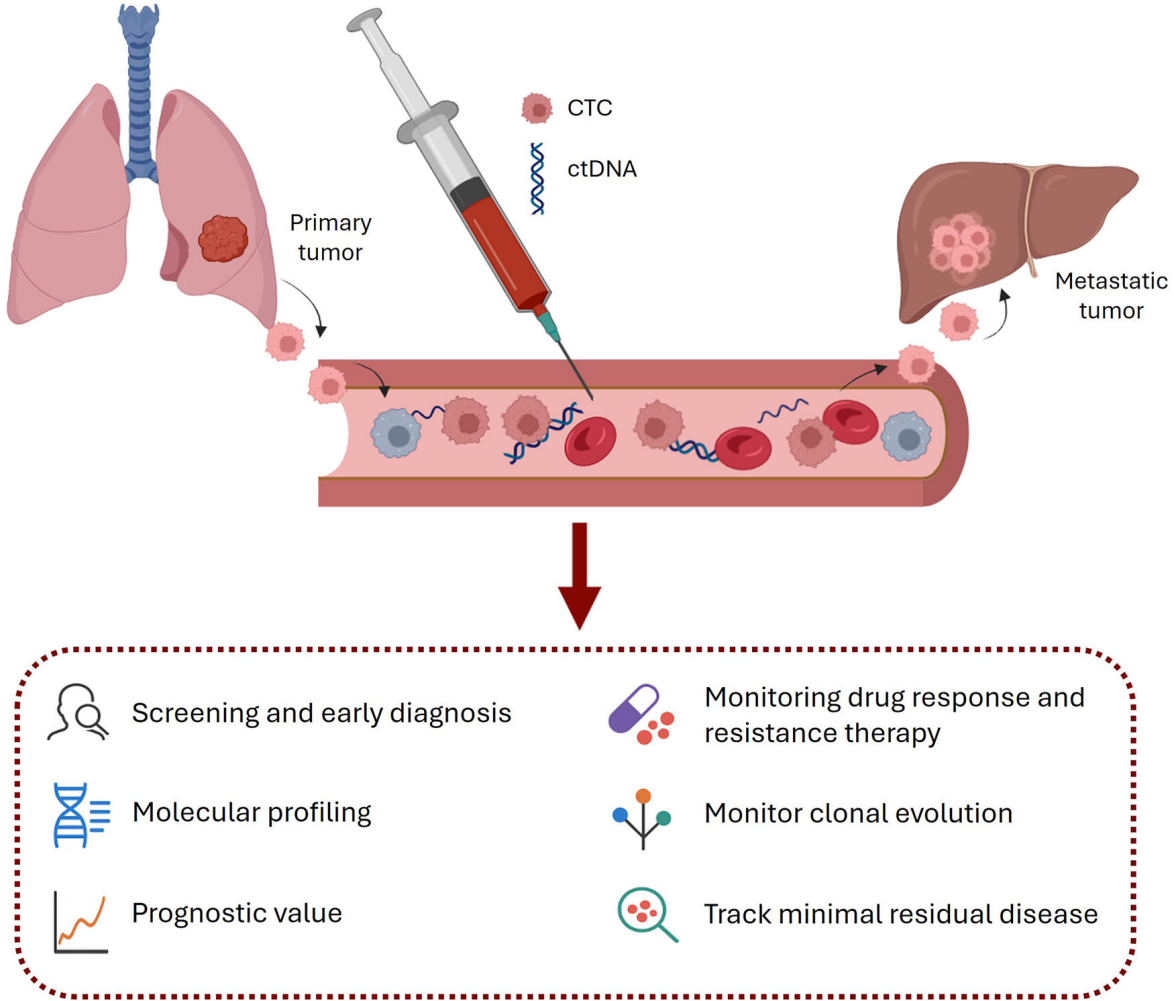
Liquid biopsy markers and their potential applications in oncology. Circulating tumor cells (CTCs) are shed from the primary tumor into the bloodstream, where they may disseminate and seed distant organs, contributing to metastasis. Circulating tumor DNA (ctDNA) originates from apoptotic or necrotic tumor cells, as well as CTCs, and enters the circulation as fragmented DNA. Analysis of these liquid biopsy components supports multiple clinical applications, including cancer screening and early diagnosis, molecular profiling, prognostic assessment, minimal residual disease monitoring, evaluation of therapeutic response and resistance, and tracking of clonal evolution over time.

Among these biomarkers, CTCs and ctDNA are the most extensively studied. CTCs are shed from primary or metastatic tumor sites, traverse the endothelium, and circulate in the bloodstream (Fig. 2). Their presence reflects ongoing tumor proliferation and dissemination, processes driven by alterations in genes involved in cell survival, invasiveness, and genome integrity ^15^.

ctDNA represents the tumor-derived fraction of cfDNA^16^. Cell-free DNA consists of extracellular double-stranded DNA fragments released into circulation predominantly through apoptosis and necrosis of both normal and malignant cells (Fig. 2), as well as from CTCs. cfDNA is typically highly fragmented due to nuclease activity and is rapidly cleared from the bloodstream, with an estimated half-life of 5 to 150 minutes, making it a highly dynamic genomic biomarker. ctDNA carries a unique genetic and epigenetic signature that mirrors its tumor of origin, enabling its distinction from background cfDNA and supporting precise detection of tumor-associated molecular features ^17,18^.

Advances in the detection and analysis of circulating biomarkers have established liquid biopsy as a powerful tool in cancer management. The biological properties of CTCs and ctDNA, particularly their ability to reflect real-time tumor dynamics, allow liquid biopsy–based approaches to provide clinically meaningful information (Fig. 2). As a result, liquid biopsy supports key applications in precision oncology, including comprehensive molecular profiling, real-time monitoring of tumor evolution, early detection of therapeutic resistance, and guiding treatment selection ^10,19^.

### Comparison with tissue biopsy and advantages

Tissue biopsy remains the gold standard for cancer diagnosis and treatment selection in solid tumors. However, it is an invasive procedure, and in certain anatomical locations (e.g., lungs or pancreas), obtaining sufficient material can be particularly challenging. Additionally, tissue biopsies are typically performed only once a tumor mass becomes detectable via imaging techniques, which may delay diagnosis and negatively influence treatment outcomes^20^. Advances in sequencing technologies have revealed a high degree of intratumor heterogeneity in many cancers^21^, highlighting a fundamental limitation of single-site tissue sampling for diagnosis, prognosis, and therapeutic decision-making ^22^.

**Fig. 3:**
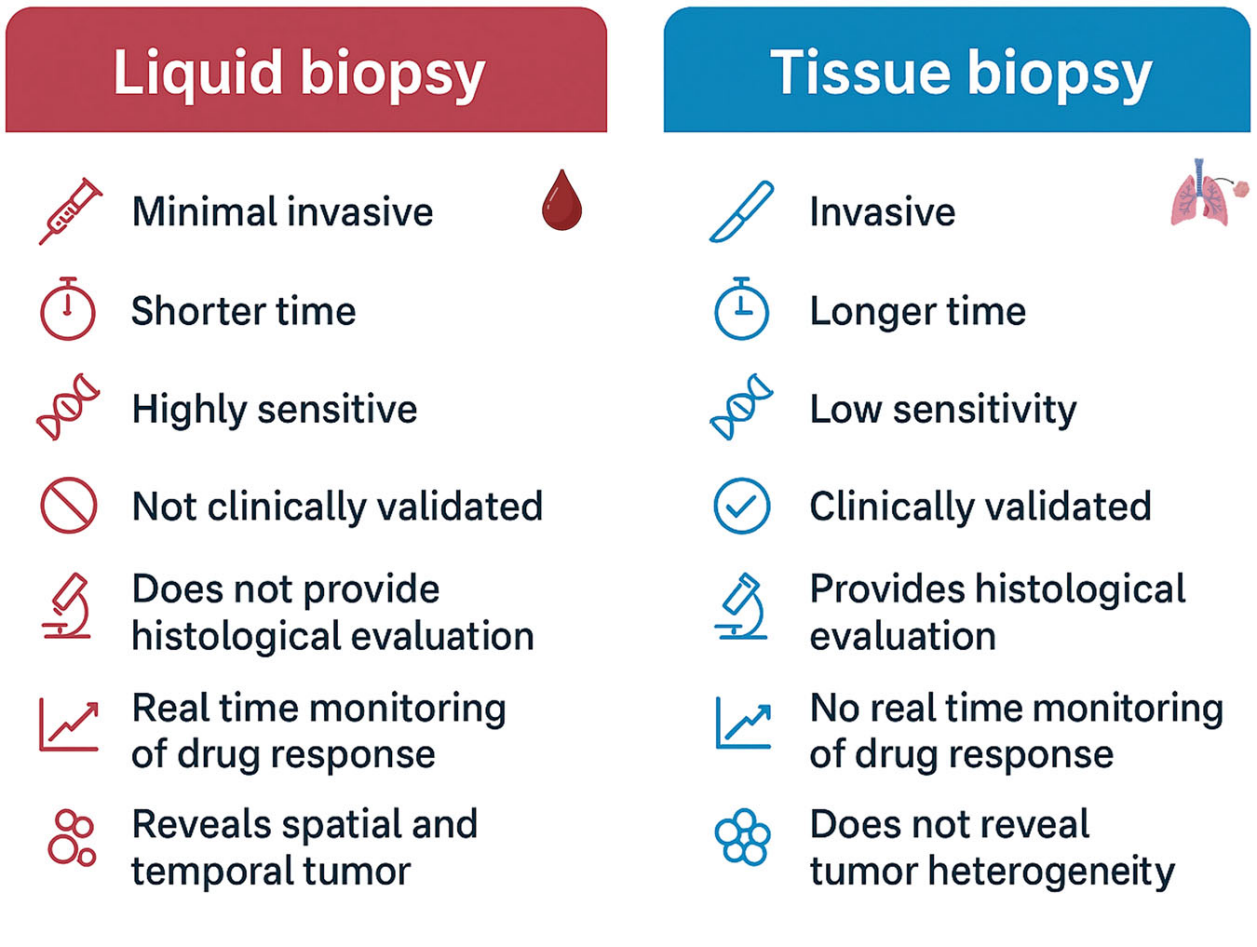
Comparison between traditional tissue biopsy and liquid biopsy. The figure contrasts key characteristics of liquid biopsy and conventional tissue biopsy. Compared to tissue biopsy, liquid biopsy is minimally invasive, faster, and highly sensitive, enabling real-time monitoring of treatment response and capturing spatial and temporal tumor heterogeneity. In contrast, tissue biopsy is invasive, time-consuming, and limited sensitivity, although it remains clinically validated and provides essential histological information.

Many of these limitations can be mitigated through liquid biopsy (Fig. 3). This minimally invasive approach enables the analysis of tumor-derived material from body fluids, offering a broader and more representative view of the genetic landscape of both primary and metastatic lesions. Liquid biopsy also permits longitudinal, real-time monitoring of tumor evolution, thereby complementing, and in some contexts surpassing, the information provided by conventional tissue biopsies ^20^.

### Use of liquid biopsy in research and clinical practice

Circulating tumor DNA has received considerable attention because it reflects the tumor’s genetic landscape^18^ and offers a unique opportunity for dynamic, real-time tumor monitoring^23^. Since plasma can be sampled repeatedly over time, ctDNA is increasingly recognized as an ideal biomarker for tracking tumor evolution. Notably, longitudinal plasma cfDNA testing has already been implemented clinically to monitor tumor dynamics and treatment response in lung, breast, and gastroesophageal cancers^24–26^. For example, somatic *EGFR* mutations can be detected in plasma cfDNA to guide the use of EGFR tyrosine kinase inhibitors in NSCLC patients ^24,26^.

However, routine implementation of liquid biopsy remains limited in many other tumor types, including glioblastoma^27^, bladder^28^, or prostate cancer^29^, largely due to pronounced genetic heterogeneity, which complicates the design of universal biomarker panels. Moreover, unlike tissue biopsy, which has undergone decades of clinical standardization, liquid biopsy assays are still in the process of analytical and clinical validation across cancer types^30,31^. This lack of broad clinical validation represents a major barrier to the widespread translation of liquid biopsy into routine clinical practice.

To address these challenges, multiple tumor-derived DNA detection and cfDNA analysis strategies have been developed. Tumor-informed approaches begin with sequencing the primary tumor to identify patient-specific mutations, which are subsequently tracked in plasma. This strategy has shown clinical utility for detecting molecular relapse and assessing therapeutic response, particularly in bladder cancer^32–34^. However, the individualized assay design increases workload, turnaround time, and cost, limiting broad implementation.

Alternatively, tumor-agnostic approaches employ standardized panels targeting the most frequently mutated genes in specific cancer types. These assays enable patient monitoring without requiring matched tumor sequencing and have demonstrated feasibility in breast^35^, colorectal^36^, ovarian^37^, and lung cancers^38,39^. Notably, in 2016, the FDA approved the Cobas EGFR Mutation Test v2 (Roche Molecular Systems, Inc.) as the first companion diagnostic test using plasma cfDNA to detect *EGFR* alterations and identify NSCLC patients eligible for erlotinib therapy^40^. Nonetheless, the applicability of tumor-agnostic approaches in highly heterogeneous cancers, such as bladder or colorectal cancer, remains limited. Fixed gene panels may fail to capture the full spectrum of molecular alterations arising during tumor progression or treatment, and detection sensitivity may be low ^41–43^.

Advances in next-generation sequencing (NGS) offer a promising solution to overcome the limited clinical validation of liquid biopsy. Broad, untargeted approaches, such as whole-exome sequencing (WES) and whole-genome sequencing (WGS), enable comprehensive detection of genomic alterations directly from plasma, without requiring prior tumor-informed profiling^44–48^. These methods are particularly relevant for genomically unstable or heterogeneous tumors that lack recurrent, targetable driver alterations. For example, brain metastases frequently differ genetically from their primary tumors, and obtaining metastatic tissue is challenging. Several studies have shown that NGS analysis of cfDNA extracted from cerebrospinal fluid of lung cancer patients with brain metastases can accurately capture the genetic profile of these lesions^49–51^, thereby guiding more effective treatment strategies.

Consequently, NGS-based comprehensive genetic profiling (CGP) via liquid biopsy represents a compelling approach for advancing molecular tumor characterization and improving clinical decision-making in oncology.

### Mutational signatures

Somatic mutations in the cancer genome are routinely analyzed using NGS to identify genetic alterations that may guide therapy and inform tumor evolution. However, not all cancers harbor actionable or predictive mutations, posing a challenge for precision oncology.

Recent advances in DNA sequencing have enabled genome-wide characterization of somatic variants that accumulate during carcinogenesis. These variants arise from endogenous processes, such as the intrinsic infidelity of DNA replication, enzymatic DNA modification, or defects in DNA repair pathways──which can be initiated or exacerbated by early driver mutations──as well as from exogenous processes, including exposure to carcinogens that induce DNA damage or chemical modifications that underlie cancer development. Together, these mechanisms generate characteristic patterns of mutations in the genome, known as mutational signatures, which provide valuable clues about the etiology and evolution of cancer ^52–54^.

Prior to the widespread use of whole genome sequencing, mutational patterns were primarily identified through the analysis of individual genes. For example, in lung cancer patients tested for *KRAS* and *TP53* mutations, cytosine-to-adenine (C > A) transversions were predominantly found in smokers, whereas cytosine-to-thymine (C > T) transversions were more common in non-smokers, suggesting a link between mutation type and carcinogenic exposure^55,56^. With the emergence of NGS, it is now possible to characterize the full diversity of mutational patterns accumulated across the cancer genome, both before and during tumor development and progression. To interpret these data, mathematical tools have been developed to extract mutational signatures from large cancer datasets and estimate the contribution of each underlying process. These approaches quantify the number of mutations attributable to each signature and assign probabilities linking individual mutations to specific mutational processes ^57–65^.

**Fig. 4:**
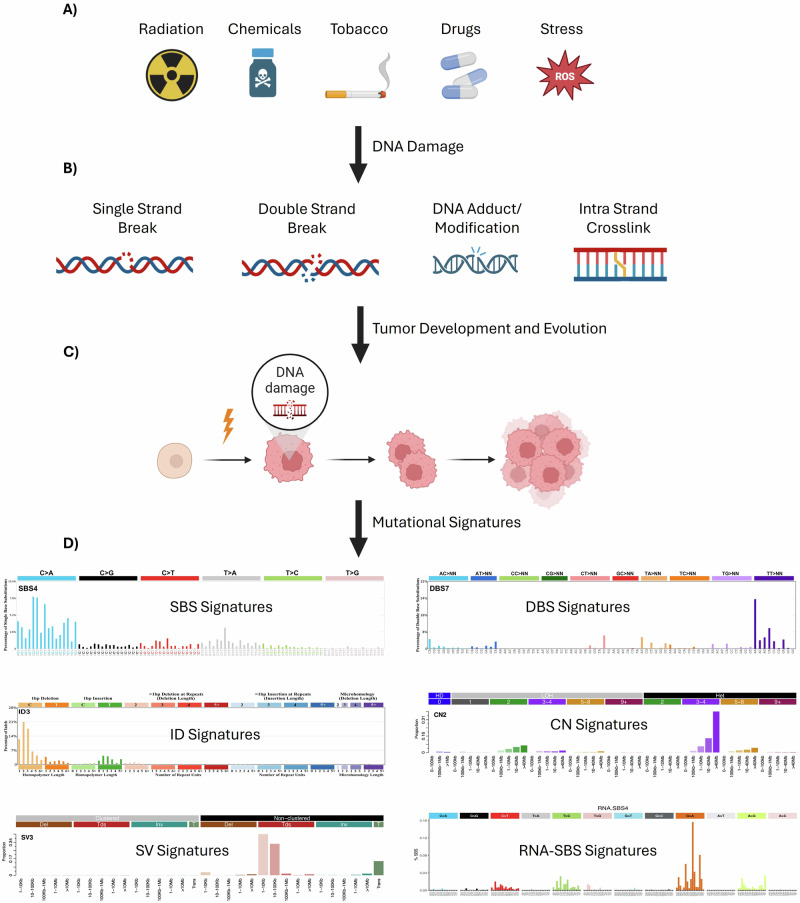
Landscape of mutational signatures across tumorigenesis. **A** The exposome encompasses the full spectrum of environmental, lifestyle, and endogenous factors encountered throughout life, including radiation, chemical exposures, tobacco smoke, therapeutic drugs, and oxidative stress. **B** These exposures induce diverse types of DNA damage (such as single- and double-strand breaks, intra-strand crosslinks, and DNA adducts or modifications) that can persist when repair mechanisms fail. **C** Replication of damaged DNA promotes the initiation, expansion, and evolution of tumor cells during tumorigenesis. **D** As tumors progress, the accumulation of somatic alterations produces distinct mutational classes, giving rise to characteristic mutational signatures. These include single-base substitutions (SBSs), double-base substitutions (DBSs), insertions and deletions (IDs), copy-number (CN) alterations, and structural variants (SVs). RNA-SBS signatures reflect analogous mutational patterns detectable at the transcriptomic level. Representative mutational signature plots are shown for illustrative purposes and do not represent patient-specific data.

Consequently, mutational signatures can reveal the sources of DNA damage contributing to tumorigenesis (e.g., tobacco smoking or exposure to specific treatments; Fig. 4A), highlight defects in DNA repair pathways (Fig. 4B), or capture the mutational processes that accumulate during tumor development and evolution (Fig. 4C) ^53,66^.

The Catalog of Somatic Mutations in Cancer (COSMIC) has established a comprehensive reference of mutational signatures, primarily derived from the Pan-Cancer Analysis of Whole Genomes (PCAWG) dataset^67^ and supplemented with exposure-specific datasets using the SigProfiler algorithm^53^. The current COSMIC catalog includes 146 defined signatures derived from over 23,000 cancer genomes. These signatures are grouped into five major classes (Fig. 4D): single base substitution (SBS; *n* = 80), doublet base substitution (DBS; *n* = 19), insertions and deletions (ID; *n* = 23), copy number (CN; *n* = 22), and structural variation (SV; *n* = 10). Each signature is annotated with information on proposed etiology, tissue distribution, and associations with other genomic features^53,66^. Despite these advances, the etiologies of 64 signatures remain unknown, underscoring persistent gaps in our understanding of cancer origins and evolution (https://cancer.sanger.ac.uk/signatures/).

In addition, COSMIC has recently incorporated five RNA-based mutational signatures (Fig. 4D). These were extracted from RNA-specific single nucleotide variants identified in 333 non-small cell lung cancer samples^68^. By excluding RNA variants that overlapped with DNA variants from matched exome data, these RNA-SBS signatures uniquely reflect RNA editing processes. The study linked these signatures to the activity of the RNA-editing enzymes ADAR and APOBEC3A, revealing previously undetected APOBEC activity in tumors. However, the etiologies of only two out of five RNA-SBS signatures are currently defined.

**Fig. 5:**
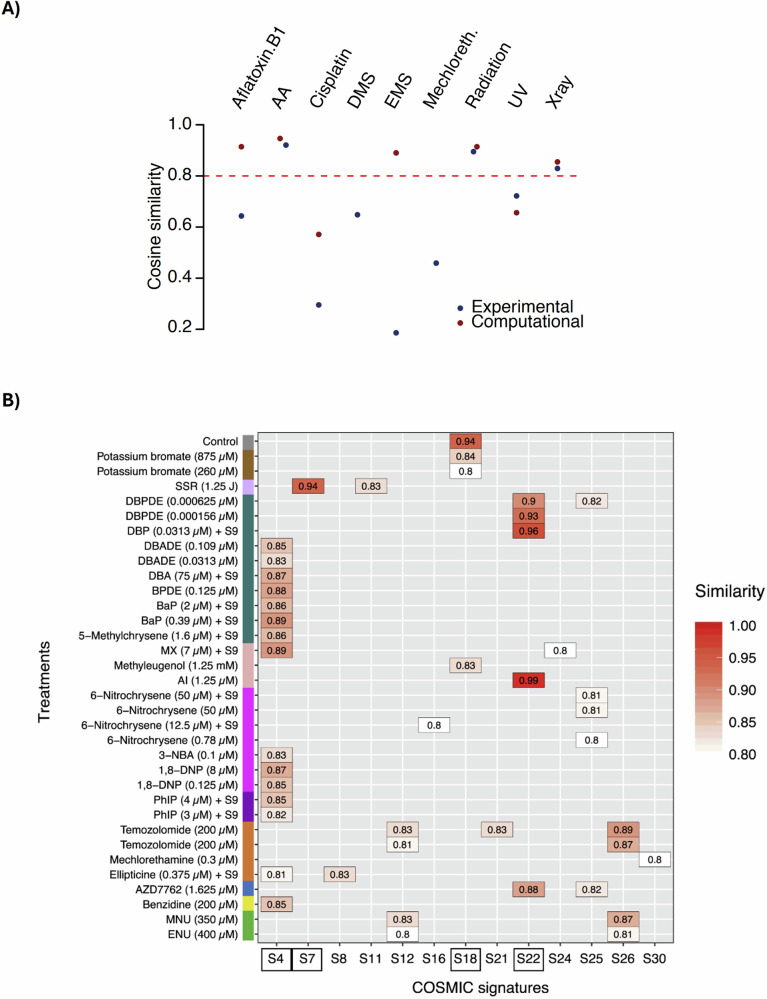
Experimental mutagen signatures across species and their correspondence to human cancer mutational signatures. **A** Cosine similarity between humanized *C. elegans* mutational signatures and both experimentally derived (blue) and computationally inferred (red) signatures observed in human cells or cancers. The red horizontal line indicates the cosine similarity threshold of 0.8, above which mutational spectra are considered closely related. Figure reproduced from Volkova et al.^69^. **B** Correspondence between COSMIC SBS signatures and substitution signatures experimentally generated in human cell models exposed to defined mutagens. Figure reproduced from Kucab et al.^70^.

Experimental mutagenesis models have further expanded our understanding of exposure-induced mutational processes beyond human cancer studies. Analysis of more than 3900 samples has identified 140 experimental mutational signatures across multiple species (*Homo sapiens*, *Mus musculus*, *Rattus norvegicus*, *Caenorhabditis elegans*, and *Gallus gallus*), enabling cross-organism comparisons of mutational patterns induced by specific carcinogens (https://cancer.sanger.ac.uk/signatures/experimental/). The broad conservation of genotoxin-derived signatures across eukaryotes reflects the conserved nature of DNA repair pathways, while differences between species, or even between human cell lines, highlight variation in metabolic activation and repair capacity. For instance, Volkova et al.^69^. used *C. elegans* whole-genome sequencing to characterize mutational processes induced by individual genotoxins and found that over 50% of *C. elegans* signatures showed low similarity to their human counterparts (cosine similarity < 0.8; Fig. 5A). Conversely, Kucab et al. ^70^. analyzed mutational signatures induced by environmental carcinogens in a human cell model and observed strong matches between many experimental signatures and COSMIC SBS signatures (cosine similarity ≥ 0.8; Fig. 5B). For example, benzo[a]pyrene (BaP) induced a signature highly similar to SBS4, enriched for C > A transversions commonly seen in lung cancers, while aristolochic acid I (AAI) produced a pattern resembling SBS22, enriched for thymine-to-adenine (T > A) transversions frequently observed in bladder cancer.

Together, these findings demonstrate that experimental human model systems are powerful tools for identifying exposure-specific mutational signatures that closely mirror those found in human cancers. Integrating signatures from both human tumors and experimental models provides critical insights into DNA damage and repair mechanisms, advancing our understanding of cancer etiology and evolution.

### Biological relevance of mutational signatures

Mutational patterns in the cancer genome represent the imprints of DNA damage and repair processes that arise during tumorigenesis and persist within tumor cells. Humans are continuously exposed to carcinogenic agents, both occupationally and environmentally, that can induce DNA adducts and generate mutations in the genome^71^. Some mutational signatures have been directly linked to specific environmental exposures, such as ultraviolet (UV) radiation (SBS7) and tobacco smoking (SBS4), whereas others reflect deficiencies in DNA repair pathways, including mismatch repair (SBS6, SBS15, SBS21, and SBS44) or homologous recombination (SBS3). Additional signatures are associated with treatment-induced mutagenesis, such as those generated by platinum-based chemotherapy (SBS31, SBS35) or temozolomide (SBS11) ^66,72^.

Interestingly, certain exogenous agents can activate endogenous mutational processes, resulting in indistinguishable mutational patterns. For example, exposure to carcinogens that induce reactive oxygen species (ROS) leads to SBS18^73^, a signature that is also observed under conditions of endogenous oxidative stress. This interplay is well illustrated in xeroderma pigmentosum (XP), a disorder characterized by defects in nucleotide excision repair. XP patients exhibit a markedly increased risk of skin cancer in sun-exposed areas due to impaired repair of UV-induced DNA damage. Skin tumors from XP patients frequently display the SBS7 signature associated with UV radiation, but also signatures linked to oxidative damage, such as SBS18. This observation suggests that UV exposure can indirectly promote oxidative stress, thereby activating additional endogenous mutational processes^74^. Together, these findings demonstrate that a single carcinogenic exposure can initiate multiple mutational processes, resulting in a mosaic of coexisting mutational signatures within the same tumor.

Characterizing the full spectrum of mutational profiles in human cancers over time can elucidate processes driving tumor development and evolution. Ashley et al.^75^ demonstrated that mutational processes differ between primary tumors and metastases, as well as among tumors within the same molecular subtype, highlighting how mutational signatures can reveal intratumor heterogeneity and disease progression in endometrial cancer. Similarly, Woolston et al.^76^ identified the emergence of a chemotherapy-induced signature (SBS17b) in colorectal cancer during treatment, illustrating how mutational profiles can serve as predictors of tumor evolutionary dynamics.

Understanding these mutational patterns offers key insights into the evolutionary history of tumors and has important clinical implications. Several studies have shown that mutational signatures can uncover DNA repair deficiencies across cancer types^75,77,78^, thereby identifying patients who may benefit from therapies targeting tumors with specific repair defects. Recognition of such patterns may further expand precision oncology opportunities through synthetic lethality or other emerging therapeutic strategies ^52^.

### Combination of liquid biopsy and mutational signatures for precision oncology

Liquid biopsy has become a pivotal tool in precision oncology. However, in several cancer types, the low abundance of tumor-derived genetic material in circulation can hinder accurate determination of the tumor molecular profile^79^. The development of highly sensitive technologies capable of detecting variants at extremely low allele frequency (as low as 0.01% VAF) has now enabled ctDNA detection across nearly all cancer stages, from early diagnosis to relapse and progression. In parallel, CGP of cfDNA using NGS-based approaches, such as WGS or WES offers a powerful strategy to uncover mutational signatures associated with specific cancer types and etiologies^53,80,81^. This approach holds particular value for tumors that are unresectable, of unknown primary, lack prior genomic characterization, or require longitudinal monitoring of tumor evolution. However, although cfDNA-based mutational signature analysis shows promise in precision oncology, no mutational-signature-based assays have yet received clinical approval.

### Cancer screening

Distinct solid tumor types often harbor characteristic mutational patterns. For example, lung cancer is associated with the tobacco smoking signature (SBS4)^82^, colorectal carcinoma frequently exhibits mismatch repair deficiency (SBS6)^83^, cutaneous tumors commonly display UV light-induced signature (SBS7)^84^, and bladder cancer is enriched for APOBEC-related mutagenesis (SBS2/SBS13)^85,86^. These patterns, therefore, carry substantial potential for predicting tumor origin.

In recent years, circulating mutational signatures have attracted growing interest as biomarkers for cancer detection (Table 1). Wang et al.^87^ demonstrated that mutational signatures represent robust diagnostic biomarkers and provide critical insights for predicting tissue of origin. Their work showed that cancer types exhibit distinct mutational patterns that improve tissue-of-origin classification beyond what can be achieved using somatic mutations alone. Furthermore, integrating somatic alterations with mutational signatures inferred from cfDNA enabled highly accurate tumor classification. In challenging cases, such as breast versus prostate cancers──two malignancies that share numerous mutated genes──mutational signatures allowed discrimination of tumor origin with 90% accuracy. More recently, Mata et al.^88^, reported that NGS-based UV signature analysis in liquid biopsy can refine the diagnosis of cutaneous malignancies of uncertain origin and guide treatment selection in patients with advanced disease. These findings highlight the diagnostic value of mutational signature analysis in cfDNA.

**Table 1:** Summary of use of circulating mutational signatures for cancer detection and diagnosis

Reference	Type of sample	Findings	Limitations	Contributions to Precision Oncology
Bruhm et al., 2023 ^91^.	cfDNA and tissue	Developed a machine-learning tool for de novo cancer detection and characterization using mutational signatures.	Small cohort size.	Provides a non-invasive and cost-effective strategy for de novo cancer detection and characterization.
Hollizeck et al., 2024 ^90^.	cfDNA	Developed *MisMatchFinder*, enabling mutational signature detection from low-coverage cfDNA WGS.	Relies on signature-fitting tools originally designed for tissue-sequencing data.	Enables cost-effective cfDNA analysis to improve cancer detection using low-coverage WGS.
Cheng et al., 2025 ^92^.	cfDNA and tissue	Developed a deep duplex WGS approach for agnostic detection of tumor-derived mutational patterns in low-level cfDNA.	Duplex sequencing captures fewer unique molecules compared with standard sequencing.	Facilitates a non-invasive and cost-effective approach for detecting circulating mutational signatures and improving cancer screening.
Mata et al., 2025 ^88^.	cfDNA and tissue	Identified UV-related mutational signatures in plasma using a computational algorithm; proposed as a biomarker for cancers of cutaneous origin.	Requires a minimum number of variants per signature; not all cutaneous carcinomas exhibit UV-associated mutagenesis.	Offers a non-invasive approach for identifying cancers of cutaneous origin, improving diagnostic refinement and treatment guidance for advanced malignancies.
Wan et al., 2022 ^89^.	cfDNA	Detected mutational signatures from low-coverage plasma WGS in cancer patients.	Lack of matched germline samples limited complete characterization of somatic signatures.	Provides a non-invasive and cost-efficient approach to support early cancer detection and assessment of cancer risk and etiology.
Wang et al., 2022 ^87^.	cfDNA and tissue	Demonstrated that mutational signatures distinguish cancer from inflammation and healthy individuals and predict tissue of origin for primary and metastatic tumors.	Limited number of mutational signatures evaluated.	Supports improved cancer diagnosis and tracing of tumor origin using a non-invasive framework.

*cfDNA* cell-free DNA, *WGS* whole-genome sequencing.

Despite these advances, many studies still require costly deep sequencing of cfDNA to achieve reliable somatic variant calling at low mutant allele fractions. To address this challenge, several groups have developed low-coverage sequencing approaches ( < 10X) to improve characterization of mutational signatures in cfDNA from cancer patients^89–91^. These methods apply mathematical algorithms to suppress technical and biological noise, coupled with machine learning to enrich tumor-derived variants. For example, one study demonstrated that the APOBEC-linked signature (SBS2), one of the most common mutational processes in human cancers, can effectively distinguish cancer samples from healthy controls across multiple cancer types^90^. The same study also identified tumor-type-specific mutational patterns in cfDNA, confirming the potential of circulating mutational signatures for cancer detection. Although low-coverage approaches still face technical limitations, they offer an attractive and cost-efficient avenue for clinical translation by enabling signature detection despite low levels of circulating tumor-derived DNA. More recently, Cheng et al.^92^ developed a new low-cost WGS approach capable of detecting tumor-derived DNA even at low cfDNA fractions. This method combines deep duplex sequencing with single-end sequencing, improving the efficiency and accuracy of genome-wide profiling. Importantly, their study demonstrated that agnostic detection of tumor-derived mutational patterns substantially expands the applicability of ctDNA monitoring in common clinical scenarios.

Collectively, these findings underscore the strong potential of circulating mutational signature analysis for cancer screening and molecular diagnostics. The integration of liquid biopsy with machine-learning-enabled mutational signature profiling provides a promising and cost-efficient framework to detect tumor-specific mutational processes. When combined with key oncogenic drivers, this approach can improve diagnostic accuracy, enhance precision oncology, and thereby improve cancer patient outcomes. However, these findings are currently supported primarily by retrospective analyses and early-stage studies, and prospective validation in large, clinically annotated cohorts will be required before clinical translation.

### Personalized treatment

Liquid biopsy-based CGP has the potential to guide precision oncology, as cfDNA can reveal tumor-specific mutational signatures that may be linked to targeted or immunological therapies. Although no studies to date have directly used mutational patterns in cfDNA to select or guide treatment, the characterization of circulating mutational signatures represents a promising strategy for therapy stratification and treatment monitoring.

Tumors exhibiting homologous recombination repair (HRR) deficiency signatures, frequently associated with BRCA1/2 mutations, as observed in ovarian, breast, pancreatic, and gastric tumors^93–96^, are particularly sensitive to platinum-based chemotherapy and PARP inhibitors. Patients harboring these HRR deficiency-associated mutational signatures could therefore be strong candidates for these therapies, potentially complementing established tissue-based assays for HRR deficiency.

Similarly, mismatch repair (MMR) deficiency generates characteristic mutational signatures marked by elevated mutation rates at short tandem repeats, leading to microsatellite instability^97^. MMR-deficient tumors demonstrate remarkable sensitivity to immune checkpoint blockade^98,99^. Reflecting this, the FDA approved pembrolizumab for any resectable or metastatic solid tumor with MMR deficiency, the first tumor-agnostic approval^100^. However, detecting MMR deficiency can be challenging, particularly in tumors with high mutational burden. A recently developed mutational-signature-based classifier, MMRDetect, reliably identifies MMR-deficient tumors and may be applicable to cfDNA analysis ^101^.

Another clinically relevant process is APOBEC-mediated mutagenesis, frequently observed in bladder and lung cancers. APOBEC-associated signatures have been proposed as biomarkers of responsiveness to immunotherapy^102^, further highlighting the therapeutic implications of mutational signature analysis.

Most patients treated with chemotherapy or immunotherapy eventually develop disease progression due to the acquisition of resistance mechanisms. Monitoring tumor evolution via cfDNA enables real-time detection of emerging resistant clones by identifying new mutations or shifts in mutational signatures. Additionally, because many chemotherapies are genotoxic, a subset of patients may develop secondary tumors resulting from treatment-induced DNA damage^103,104^. Notably, cisplatin-based chemotherapy, a widely used platinum-based therapy across bladder, breast, ovarian, and other cancers^105–107^, is associated with a characteristic mutational signature that has been consistently observed across multiple patient cohorts with advanced or metastatic disease^103,104,108–111^. Detection of this cisplatin-related signature in cfDNA offers a potential means to identify therapy-induced mutagenesis during treatment.

In summary, mutational signatures detected in cfDNA may help refine therapeutic decision-making and enable real-time monitoring of tumor evolution in response to therapy, ultimately supporting more personalized and adaptive treatment strategies in oncology. Finally, bridging the gap between discovery and clinical deployment will require standardized signature extraction pipelines, analytical validation in low-input plasma samples, definition of clearly actionable clinical endpoints, and demonstration of clinical utility in prospective trials.

### Potential clinical decision points informed by mutational signatures

Although mutational signatures are not yet used as standalone clinical biomarkers, they may realistically inform several specific clinical decision points when integrated with existing genomic and clinical data. First, mutational signatures may support therapy eligibility, particularly in cases where DNA repair deficiency is present without identifiable driver mutations, such as homologous recombination deficiency (SBS3) or mismatch repair deficiency (SBS6, SBS15, SBS21, and SBS44) detected at the process level rather than through single-gene alterations alone.

Second, mutational signatures may contribute to treatment sequencing, for example by identifying tumors with ongoing APOBEC- or platinum-induced mutagenesis (SBS2/13 or SBS31/35) that may be more prone to rapid resistance evolution, thereby informing earlier treatment intensification or alternative therapeutic strategies.

Third, longitudinal monitoring of circulating mutational signatures may enable resistance surveillance, as shifts in signature activity could precede the emergence of overt resistance mutations detectable at the variant level.

Finally, mutational signatures may support risk stratification, particularly in minimal residual disease settings, where persistent or therapy-induced mutational processes in cfDNA could indicate ongoing genomic instability and increased relapse risk.

Importantly, mutational signatures are likely to function as complementary biomarkers, rather than replacements for established markers, such as tumor mutational burden, microsatellite instability, or homologous recombination deficiency scores. Their added value lies in providing a mechanistic, process-level context that may refine the interpretation of existing biomarkers and support more adaptive precision oncology strategies.

### Challenges and limitations

Mutational signatures, derived from somatic alterations accumulated during tumor development and evolution, are often complex and multifactorial. They may reflect combinations of environmental exposures, endogenous cellular processes, multiple mutational sources, or even unknown causes^72^, making it difficult to determine the specific origin of the mutations that characterize a tumor.

The identification of mutational signatures in liquid biopsy introduces additional challenges. First, some tumors shed insufficient DNA or tumor cells into the bloodstream, limiting the detection of tumor-derived alterations^112^. Second, many mutational signatures still lack a defined etiology because the biological processes driving their formation remain unclear. In addition, some signatures may arise from sequencing artifacts, complicating the interpretation of cancer origins^113^. Third, variability among sequencing platforms can influence detected mutational patterns, as each NGS technology introduces specific biases and technical artifacts during library preparation that ultimately affect signature extraction ^114^.

Moreover, the clinical translation of mutational signatures remains limited due to the lack of analytically and clinically validated methods for several signatures. Although UV radiation- or tobacco smoking-associated signatures have been strongly associated with cutaneous and lung tumors, respectively, these associations are not universal, as only a subset of melanomas and lung cancers exhibit these patterns. Standardized protocols, reference standards, and interlaboratory concordance studies may therefore be required to support robust analytical validation of mutational signatures.

Approaches developed to detect mutational patterns in cfDNA often rely on machine learning to improve sensitivity^89,91^, but these methods require extensive training and validation using large datasets^115^, which may limit their immediate clinical applicability. Other tools that integrate mutational signatures from plasma sequencing data^90^ depend on fitting algorithms originally designed for tumor tissue sequencing data^116,117^, and may show inconsistent performance when applied to cfDNA.

Another major challenge in detecting tumor-derived mutations is the co-occurrence of somatic mutations in leukemia-related genes within cfDNA^118,119^, which may confound tumor-specific signal interpretation. These mutations arise from clonal hematopoiesis of indeterminate potential (CHIP), defined as the expansion of hematopoietic stem cells harboring somatic mutations without an underlying hematologic malignancy. CHIP is strongly associated with advanced age^120,121^, thereby contributing to age-associated SBS patterns (e.g., SBS5) detected in cfDNA samples from cancer patients and potentially altering result interpretation. In addition, prior cancer therapies or tobacco exposure may induce mutagenesis in hematopoietic cells^122^, introducing treatment- or tobacco-related mutational patterns into cfDNA that further complicate tumor-specific signature attribution. Sequencing of matched white blood cells can help identify CHIP-derived mutations and distinguish them from tumor-derived mutations in cfDNA ^123,124^.

Expanding datasets of matched tumor, normal, and plasma sequencing samples across diverse cancer types will be crucial to developing algorithms specifically optimized for cfDNA, thereby improving mutational signature detection in liquid biopsy. Ultimately, research aimed at clarifying the etiology of tumor mutational patterns, together with prospective studies associating mutational profiles with treatment selection and patient outcomes, will provide critical insights into cancer development and evolution and support further advances in precision oncology.

## Conclusions

Integrating liquid biopsy with mutational signature analysis offers a powerful conceptual and translational framework to deepen our understanding of cancer origins and evolution. Together, these complementary approaches hold significant potential to enhance early cancer detection, refine diagnostic accuracy, and inform personalized therapeutic strategies.

Despite this promise, the clinical implementation of circulating mutational signature analysis remains at an early stage. Most evidence to date is derived from retrospective and proof-of-concept studies, underscoring the need for prospective, clinically annotated trials that directly evaluate clinical utility across screening, treatment stratification, resistance monitoring, and minimal residual disease settings. In parallel, regulatory and reimbursement frameworks will need to evolve to accommodate process-level biomarkers that extend beyond single-gene alterations and variant-centric assays.

Looking ahead, advances in artificial intelligence and integrative modeling are expected to play a central role by enabling the joint analysis of mutational signatures with variant-level genomic, epigenetic, and clinical data. These developments may facilitate the adoption of dynamic, non-invasive biomarkers and further expand the scope of precision oncology.

## Data Availability

No datasets were generated or analyzed during the current study.
